# Connecting the Mind–Body Split: Understanding the Relationship between Symptoms and Emotional Well-Being in Chronic Pain and Functional Gastrointestinal Disorders

**DOI:** 10.3390/healthcare5040093

**Published:** 2017-12-05

**Authors:** Line Caes, Alex Orchard, Deborah Christie

**Affiliations:** 1 Division of Psychology, Faculty of Natural Sciences, University of Stirling, Stirling FK9 4LA, UK; 2 UCLH NHS Foundation Trust, Child and Adolescent Psychological Services, London NW1 2PQ, UK; alexandra.orchard@nhs.net (A.O.); Deborah.christie2@nhs.net (D.C.)

**Keywords:** pain, distress, mind-body split

## Abstract

Paediatric chronic conditions, e.g., chronic pain and functional gastrointestinal disorders, are commonly diagnosed, with fatigue, pain and abdominal discomfort the most frequently reported symptoms across conditions. Regardless of whether symptoms are connected to an underlying medical diagnosis or not, they are often associated with an increased experience of psychological distress by both the ill child and their parents. While pain and embarrassing symptoms can induce increased distress, evidence is also accumulating in support of a reciprocal relationship between pain and distress. This reciprocal relationship is nicely illustrated in the fear avoidance model of pain, which has recently been found to be applicable to childhood pain experiences. The purpose of this article is to illustrate how mind (i.e., emotions) and body (i.e., physical symptoms) interact using chronic pain and gastrointestinal disorders as key examples. Despite the evidence for the connection between mind and body, the mind–body split is still a dominant position for families and health care systems, as evidenced by the artificial split between physical and mental health care. In a mission to overcome this gap, this article will conclude by providing tools on how the highlighted evidence can help to close this gap between mind and body.

## 1. Introduction

Having a chronic illness greatly increases the risk of experiencing psychological distress [1,2]. Living with a long-term condition can have an impact on social development, family relationships, education and age-related developmental tasks affecting identity, resilience and self-esteem as well as future hopes and dreams [3,4,5]. Chronic conditions that present with long term fatigue, pain and abdominal symptoms represent a substantial proportion of referrals to rheumatology, gastroenterology and general adolescent medicine clinics. Approximately a third of adolescents complain of substantial fatigue 4 or more times a week. Symptoms frequently observed across various paediatric chronic conditions can range from mild to severe fatigue, transient aches and pains such as headaches or abdominal pain and/or presentations of complete inability to move, eat or speak [6].

Symptoms may be connected to an underlying diagnosis (e.g., Ehler’s Danlos, rheumatoid arthritis, inflammatory bowel disease (Crohn’s disease and ulcerative colitis)). Whilst changes in structure or biochemical interaction can be detected in young people with a medical diagnosis there are a number of young people that present with pain as the primary disorder (e.g., tension headache, functional abdominal pain, widespread musculoskeletal pain) or pain associated with fatigue or nausea that are classified as a functional or medically unexplained [7]. These functional disorders demonstrate similar reciprocal relationships between physical symptoms and emotional well-being. In this article, we will focus on two common paediatric chronic illnesses, chronic pain and functional gastrointestinal disorders (FGIDs), to illustrate the mind–body connection. While the two conditions will be discussed as distinctive conditions, it is important to keep in mind that functional abdominal pain is in many cases part of the FGID diagnosis. Therefore, some overlap in the discussion of the underlying mechanisms of the two conditions is unavoidable.

## 2. Definitions

Chronic pain is typically defined as “pain lasting longer than three months or beyond the expected healing time” [8]. Chronic pain without an identifiable organic basis occurs in 4–15% of the normative adolescent population [9]. A recent systematic review on the epidemiology of chronic pain in children revealed a prevalence between 11% and 38%, with headache (8–83%), musculoskeletal pain (4–40%) and abdominal pain (4–53%) the most frequently reported types of pain [10]. 

Functional gastrointestinal disorders (FGIDs) are the most common paediatric diagnoses for recurrent abdominal pain in children and young people [11,12]. They are described as disorders of gut-brain interaction, which cannot be attributed to another medical condition after appropriate assessment. Pathophysiological processes include pain, oversensitivity to typical gut/internal organ activity, changes in central nervous system processing, intestinal contractions, gut microorganisms, immune and/or mucus function lasting for several months [13,14].

FGIDs are postulated to involve genetics, social learning, psychology, biology, immunology, contextual and individual factors. The multiple pathophysiological processes are difficult to identify using modern medicine and their interplay is not yet fully understood. International consensus and co-operation therefore developed a range of data-driven, internationally agreed criteria to identify FGIDs based on patient report of symptoms rather than findings from medical investigations, called the Rome Criteria [13,14]. As a result, clinicians are encouraged to understand support seeking and symptom descriptions within the family’s societal expectations and cultural values [13,15,16,17]. 

### 2.1. Prevalence

Independent of aetiology, higher prevalence rates of chronic pain are observed in girls and older children [10]. Girls also meet the criteria for a FGID diagnosis more frequently than boys across cultures [12]. FGID prevalence estimates vary depending on age, gender and cultural understanding of gastrointestinal symptoms [12]. Highest community and primary care incidence is reported in infants and toddlers (27–38%), followed by adolescents (8–17%) [14,18,19]. Increased prevalence is seen in higher socio-economic, western cultures compared to lower socio-economic and/or eastern cultures [13,20,21], perhaps because some FGID symptoms are not viewed as an illness or requiring support from healthcare professionals in every culture [17]. Further large-scale epidemiological studies are required to understand the effects of socioeconomic status [22].

### 2.2. Living with the Symptoms

While many children function well despite the pain, approximately 3% are significantly disabled by the pain and accompanying symptoms, which leads to negative consequences for the child and their family and requires intensive treatment [23]. In FGIDs, patients report distressing and embarrassing symptoms including, pain, diarrhea or constipation [12,13,24].

Chronic pain, FGIDs and associated symptoms interfere with daily functioning manifested by impaired sleep patterns, impaired academic, physical, and social functioning and lower quality of life [25,26,27,28,29,30,31,32]. They have also been associated with increased psychological distress, break down in family and external relationships, as well as more primary to tertiary medical consultations [11,33,34,35,36].

## 3. Connections between Mind and Body

### 3.1. Evidence within the Context of FGID

Currently, the biopsychosocial model, characterized by the amalgamation of biological, psychological, social/cultural and developmental factors, is the most widely accepted explanation of both functional and medical disorders [13,33,37,38].

This is well illustrated in FGID. Unlike other peripheral organs the digestive tract has its own intrinsic nervous system linking the brain (hypothalamic-pituitary adrenal axis; HPA), autonomic and central nervous systems [39]. This reciprocal “gut–brain axis” has more interconnecting neurons than any other parts of the body [40]. FGIDs are thought to perpetuate through dysregulation along this multi-directional loop [41]. For example, neurological changes in the HPA are suggested to increase sensitivity to typical gut sensations, causing them to be experienced as painful. Pain pathways can thereby become activated at lower levels, explaining their heightened activity in young people with FGID diagnosis compared to healthy controls [27].

Beyond the clear neurological connections, living with embarrassing and/or painful symptoms also has the potential to contribute to poor body image, bullying or mood difficulties, including anxiety and depression, which can affect psychological and social well-being [42,43]. These difficulties might reduce a child’s capacity to engage in treatment that helps manage their condition, as well as adversely affect gut symptoms through reciprocal links with the HPA [44]. 

When emotions are involved this is thought to amplify or reduce GI sensation perception and processing via the HPA, influencing how strongly or weakly sensations in the gut are experienced [45]. As a result, when negative emotions and appraisals are present, e.g., anger, worry, hypervigilance and catastrophising of pain, GI and pain symptoms tend to be reported as more severe [35,46]. It is also important to consider that reduction in FGID or pain symptoms does not always resolve emotional difficulties [47].

Taken together, the experience of pain, gastric or otherwise, does not only induce a risk of heightened distress, evidence is accumulating in support of a reciprocal relationship between pain and distress. Psychological distress may also undermine the immune system, thereby intensifying the effects of physical illness by increasing the production of stress hormones such as cortisol [48]. 

For instance, in the context of FGID, stress has been identified as one of the largest predictors of FGID symptoms, linked to onset, maintenance and poorer recovery rates [46]. A possible explanation for this association could be because psychological distress can activate the HPA, which is thought to modulate gut immune and endocrine responses, changes in gut micro bacteria and colon movements [49]. In FGID such responses and changes are thought to trigger increased activity between gut nerves and contribute to pain symptoms, through an amplified gut to brain/HPA loop [27,50,51]. Changes in gut microbiology have additionally been suggested to affect mood [52]. Thus, reciprocal links between mind and body are at the heart of understanding and treating FGIDs [13,32]. 

### 3.2. Evidence within the Context of Chronic Pain

There is also increased vulnerability and prevalence of anxiety and depressive symptoms (i.e., distress or internalising symptoms) and post-traumatic stress disorders in children with chronic pain [33,37,38,53]. Importantly, compared to pain-free children, children with chronic pain are more likely to have seen a mental health professional and the presence of internalizing symptoms is related to more frequent medical visits, thereby challenging the traditional segregated care services for physical and mental health disorders [33]. Importantly, anxiety and depressive symptoms can be predictive of recurrent pain trajectories characterised by persistently high and increasing levels of pain [54]. For instance, higher levesl of anxiety and depression symptoms in early adolescence (age 10–11) are associated with higher frequency of headaches across adolescence [54]. Evidence is accumulating to understand the underlying mechanisms of these links, with a recent study highlighting the crucial role of poor sleep quality in explaining the reciprocal association between chronic pain and post-traumatic stress syndrome [55].

Beyond frequent co-occurrence of distress and chronic pain during childhood, paediatric chronic pain experiences also substantially increase the risk of developing a mental health disorder in adulthood regardless of whether the pain symptoms persist into adulthood [53,55,56,57]. Indeed, childhood experiences of abuse (emotional, sexual and/or physical) are documented in adults diagnosed with painful functional disorders, such as FGIDs, fibromyalgia, headaches, pelvic and chronic pain [48]. Such findings further support reciprocal links between mind and body.

The growing evidence on the reciprocal relationship between pain and distress is the key principle of the fear avoidance model (FAM), which has recently been applied to paediatric chronic pain experiences [58]. The FAM postulates that catastrophic interpretations of pain experiences induce pain-related fear and avoidance of pain-inducing activities, which gives rise to anxiety and depressive feelings. In turn, feelings of anxiety and depression are related to more pain-related disability, which can further intensify the pain experience [59]. Evidence for the applicability of this fear avoidance model in explaining paediatric pain experiences is growing, with slight modifications suggested to account for different patterns for anxiety compared to depression, and depending on developmental status [58]. For instance, large-scale studies in children and adolescents with chronic pain showed that higher levels of pain-related anxiety are associated with more functional disability as well as more perceived social impairment [60,61]. A longitudinal exploration of this association also revealed that general levels of anxiety in early adolescence (i.e., around age 13), are related to higher levels of pain-related disability and pain-related anxiety in late adolescence [62]. However, the link between depression and pain does not seem to follow the predictions from the FAM: a direct link between depression and pain catastrophizing as well as pain-related fear, rather than explaining depression via activity avoidance tendencies, revealed the best fit [58].

## 4. Reciprocal Relation between Parents and Children

Parents play a crucial role in childhood chronic illness; therefore it is important to not only consider child distress but also the reciprocial relations between parental and child functioning. Although, adolescence can be considered a time of individuation [63], young people often continue benefiting from familial support. The burden of caring for a child with chronic pain or FGID may impact on parents’ capacity to cope and support their child. Parents commonly report high levels of parental stress, anxiety, depressive symptoms, marital problems, and restrictions in personal, social and family activities [30,34]. For instance, Jordan, Eccleston and Osborn (2007) found that parents caring for a child with chronic pain report a fundamentally and unexpectedly changed life characterized by the struggle to adapt to a life filled with uncertainty, fear, distress and loss [64]. Recent evidence corroborates these findings while highlighting the multidimensional nature of parental experiences by identifying two parental narrative styles of their child’s chronic pain experiences: distress (i.e., negative emotion and unresolved orientation towards pain diagnosis) vs. resilience narratives (positive emotions and resolved orientation towards pain diagnosis) [65]. These parental experiences of distress can in turn negatively influence the child’s functioning, through the impact on parental behaviour. In particular, parents reporting high levels of distress when their child is in pain have found to engage more in protective behaviours (e.g., reassuring, comforting, request to stop pain-inducing activities [66,67,68,69], which are associated with increased child pain, distress, functional disability [70,71,72] and use of healthcare [11,73]. This bidirectional, relational process whereby caregiver’s desire to help resutls into negative consequences for the child has also been referred to as miscarried helping [74]. Interestingly, a recent investigation of miscarried helping in the context of paediatric chronic pain revealed a low agreement between parental and adolescent report of parental miscarried helping, while both reports were associated with poorer family functioning, parental protective behaviours and child depressive symptoms [74]. Attempts to understand which families are at risk for negative consequences due to child pain revealed that heightened feelings of parental distress, and associated engagement in protective behaviours, are most prevalent in parents who catastrophize about their child’s pain [65,66,67,68].

**Figure 1:**
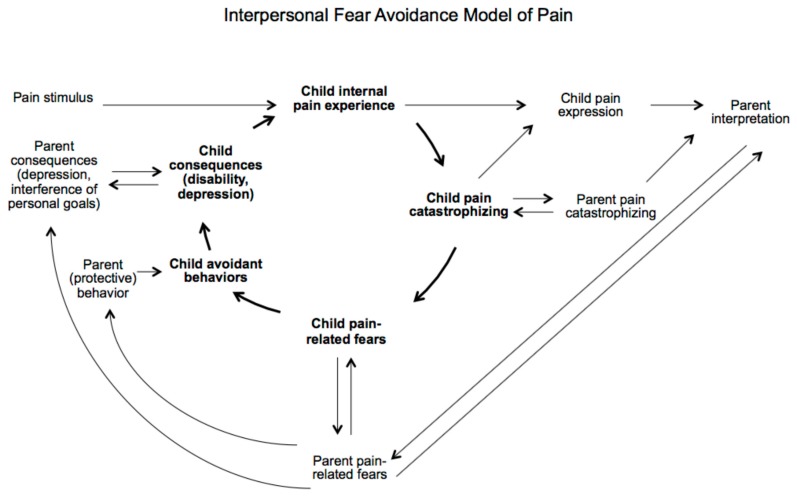
The interpersonal fear avoidance model (IFAM). Reproduced with permission from Goubert and Simons (2013) [75].

Taken together, these findings support an interpersonal application of the fear avoidance model (IFAM), proposed by Goubert and Simons (2013) [75]. See Figure 1 for a graphical overview of the IFAM. While the IFAM model is proposed within the context of chronic pain, the patterns proposed within the IFAM can be observed in the context of FGID, with poorer day-to-day functioning seen in children whose parents engage in protective behaviours [69]. Further highlighting the reciprocial influence between parents and children, poorer outcomes in FGID correlate with increased displays of pain and FGID diagnosis in parents; whether linked through genetics, social learning or other factors is unclear [76,77].

As illustrated within the IFAM, the influence of parental responses on child functioning is however complex, with recent evidence pointing to the moderating role of child characteristics. Children reporting high levels of distress or catastrophizing or low levels of self-worth or academic competence seem more vulnerable to the maladaptive impact of parents’ protective responses [70,72,78]. A high percentage of children with functional disorders have had a singular focus on academic and other achievements and are described as good, helpful and compliant, prior to illness [79]. Symptoms may then arise to reverse, shape or delay unwanted changes in relationships at times of transition, acting as a protective and adaptive mechanism and way out of an ‘intolerable predicament’ [80]. In a similar way Griffiths and Griffiths (1994) suggest symptoms develop as a way out of ‘an unspeakable dilemma’ [81]. For one reason or another these young people find it difficult to express distress through speech, therefore their bodies effectively do the talking for them [82]. 

While the majority of the above discussed evidence focusses on negative influences between parents and children, parents can also be a resource for children to enable engagement in treatment, developing coping styles that increase tolerance of symptoms and support engagement in daily life. For FGID it is suggested such strategies, sometimes enhanced or developed through psychological intervention with children and/or parents, can reduce HPA activation, thereby reducing symptoms [69,83,84]. It is also the development of trusting relationships, education and support from doctors that can positively influence outcomes for parents and their children [13,24].

## 5. Closing the Mind and Body Split

The mechanisms underpinning bodily expression of emotional distress have long been debated and are still poorly understood. Descartes proposed that the mind and the body functioned independently of one another and calls for an integrated or holistic approach to managing psychological and physical health did not take hold until the introduction of the biopsychosocial model of health, disease and illness in the 1970s by Engel [85,86].

This mind body split can be a dominant position for many families and clinicians. Anxiety experienced by families, which might perpetuate FGID and chronic pain symptoms, can relate to extensive testing contributing to fears something “dangerous” has been missed. It can contribute to contrasting views between patients, their families and clinicians. Clinicians may view functional diagnoses as more benign than their organic based cousins. Limited medical treatments and poorer outcomes for young people with functional diagnoses can prompt referrals to mental health professionals for help managing the physical and emotional symptoms in order to reduce the impact of the condition and improve quality of life [27,87]. In contrast parents and children may struggle to include psychological explanations in their understanding of the problem [88]. Many young people are categorical in their denial that there is any emotional component to their physical difficulties. Equally, it can be hard for medical professionals to see the importance of their ongoing involvement helping to indirectly reduce anxiety and psychological distress by keeping an eye on the ‘body’, which can help to improve outcomes in functional disorders [89].

Overcoming this mind body split is also made difficult by the artificial split between physical and mental health care in our current health care system. When repeated investigations prove fruitless and young people are referred to psychological services, this can be experienced as shifting the search for both cause and cure on to the individual and family functioning. The referral may be strongly resisted by the family making it difficult to establish positive family/team alliances. Families can become defensive perceiving the treatment team as blaming and critical. If not introduced carefully and at the right point in family’s journey’s more traditional psychological therapies may also be unhelpful. Psychodynamic approaches which look for underlying family conflict are often resented, while traditional psychological approaches like cognitive-behavioural therapy may not fit for the young person who cannot see a link between their thoughts and the external experience of pain and/or fatigue.

## 6. Case Example

In our Paediatric psychological services, within a large teaching hospital, we have noticed the importance of mind and body being brought together through multidisciplinary approaches, for both “functional” and “organic” disorders. This can be important in dispelling the notion that chronic pain or FGID symptoms are “all in my head” and in allowing the development of trusting relationships, which can help reduce fear and anxiety, improving engagement in treatment and rehabilitation.

A typical referral from our medical colleagues who see young people with chronic pain and/or FGID can come in the context of exhaustive testing to “rule out medical causes”. “Jan”, aged 14, was referred with a diagnosis of “chronic pain” and “visceral hypersensitivity of the gut-brain axis” (FGID). She was described as bright and able but struggling to attend school (50% attendance) because of unpredictable bouts of pain and diarrhoea. Having had initial assessments and a range of tests with both rheumatologists and gastroenterologists, she was now being referred for psychological support, with medical follow-up in one year. During initial conversations Jan was unsure why she had been referred to psychology but had very positive things to say about our rheumatology and gastroenterology colleagues. She and her mother could not make sense of how psychology could “fix” a physical problem but both agreed the current situation was not good enough as Jan really wanted to get back to school and spend time with friends.

After a few sessions of psychological support over several months Jan was not progressing either in returning to school or re-engaging in day-to-day activities she enjoyed. She described anxiety about doing more harm than good in trying day-to-day activities and frustration that pain was still around. Jan found herself doing less and less until she was barely able to get out of bed. 

Jan described the situation as improving following appointments with her doctors who talked through her conditions and reassured her mother they had not missed anything. Medication and rehabilitation options were discussed and as a result Jan saw a dietician and identified what foods she might eat to help her gastric symptoms. Jan began infrequently seeing a physiotherapist who helped her break down activities into manageable chunks and know when to push herself and when to rest. In this multidisciplinary context, a solution-focused psychological approach began to help Jan rediscover aspect of herself and interests that pain, worry and embarrassing symptoms had invited her to forget. She described confidence to try new things and re-engage in day-to-day life increasing. 

Using a non-intensive multidisciplinary approach (physiotherapy, psychology, gastroenterology, rheumatology and dietetics) Jan and her mother went from barely being able to leave the house, frequently visiting A and E, and repeatedly calling professionals to engaging in a largely self-directed rehabilitation programme, spending time with friends and beginning to return to school. They describe the importance of “knowing the hospital are there” to have the “courage and strength” to know how to push through and not be afraid of pain. Jan also began to make her own mind–body links. This example illustrates some of the literature around supporting young people with FGID and chronic pain.

## 7. Promoting the Mind and Body Working Together

As the case example illustrates, treatment approaches with good outcome rates include a close liaison between mental health and paediatric professionals, an attitude of belief in the child from staff, as well as moving the family towards a more multidisciplinary understanding of the illness [82,90,91]. For instance, a physical rehabilitation model designed to support both physical and psychological recovery [92,93], explicitly acknowledges the reality of the symptoms, emphasizes the necessary involvement of both mind and body in the recovery process, but allows young people to ‘save face’ through promoting physical recovery as the primary goal [94]. Families also feel reassured that symptoms will still be monitored. Given that up to one-third of ‘functional’ conditions are ultimately found to have an organic problem this is an essential part of the treatment programme [95]. 

Cognitive-behavioural therapy (CBT), which links thoughts, feelings and behaviours, has been incorporated into rehabilitation approaches including activity scheduling, pacing, establishment of a sleep routine, modification of negative and unhelpful thinking and relapse prevention. There have been a number of very detailed and comprehensive reviews showing the benefits of CBT, hypnosis and mindfulness amongst other psychological therapies for a variety of chronic pain syndromes and FGIDs [69,82,96,97,98,99,100]. 

Whilst CBT and graded exercise therapy (GET) are reported as effective, there is also emerging evidence about how subgroups of patients respond to different interventions that include dietetic input [101]. There is however a lack of evidence for the additive or combined effects of interventions when more than one therapy is used [102]. Ecclestone, Malleson, Clinch, Connell and Sourbut (2003) report a multidisciplinary treatment package incorporating CBT which found significant gains in physical performance, reduction in emotional distress and increased attendance in education, but it remains unclear to what extent the incorporation of CBT increased effectiveness of the treatment [103]. 

## 8. Conclusions

Historically, mind and body became separated in the medical literature and despite more recent biopsychosocial and fear avoidance models working towards reintegration, this historic split is still reflected in service design, as well as the beliefs of families and clinicians. Common paediatric functional diagnoses such as chronic pain and functional gastrointestinal disorders provide examples of the reciprocal links between mind and body. The multifaceted natures and wide-reaching effects of these diagnoses suggest the need for coordinated multidisciplinary input. 

As illustrated in this article, mind and body have symbiotic relationships, and so we endorse the benefits of mental health and paediatric services entering into their own reciprocal and supportive associations in order to provide effective care. Growing evidence points towards the pivotal nature of relationships between family members, multidisciplinary team members as well as between professionals and families. These can promote trust, understanding and rehabilitation by reducing fear and associated avoidance so that all concerned can engage in recovery. Appreciating the integrated nature of mind and body further enables children and families to work with clinicians to reduce the impact of functional disorders on day-to-day life and progress towards wellness.
